# New-onset atrial fibrillation in patients with acute hypercapnic respiratory failure requiring noninvasive ventilation

**DOI:** 10.1183/23120541.00605-2025

**Published:** 2025-12-22

**Authors:** Hani Essa, Ashwin Balu, Yusra Amanullah, Dileep Duvva, Hassan Burhan, Frederick Frost, Ari Manuel, Ingeborg Welters, Gregory Y.H. Lip

**Affiliations:** 1 Institute of Life Course and Medical Sciences, Department of Cardiovascular and Metabolic Medicine, University of Liverpool, Liverpool, UK; 2 University Hospitals of Liverpool Group, Royal Liverpool University Hospital, Liverpool, UK; 3 Liverpool Centre for Cardiovascular Science at, University of Liverpool, Liverpool John Moores University, and Liverpool Heart and Chest Hospital, Liverpool, UK; 4 Department of Clinical Medicine, Aalborg University, Aalborg, Denmark; 5 Medical University of Bialystok, Bialystok, Poland

## Abstract

**Introduction:**

Atrial fibrillation (AF) and COPD are the most common cardiac arrhythmia and chronic lung conditions worldwide. Exacerbations of COPD can be associated with acute hypercapnic respiratory failure (AHRF). It is unclear if new-onset AF (NOAF) during hospitalisation with AHRF influences long-term outcomes.

**Methods:**

We conducted a retrospective cohort study using TriNetX, a global federated health research network. Patients ≥18 years old and hospitalised with a known diagnosis of COPD and new AHRF requiring noninvasive ventilation (NIV) were divided into two cohorts based on the development of NOAF within 7 days of AHRF. After propensity score matching (1:1), there was a total of 14 213 patients in each group. Outcomes were recorded at 1 year from the index admission to hospital. The outcomes of interest were all-cause death, re-admission, stroke, myocardial infarction, composite embolic end-point (acquired absence of limb, acute vascular disorders of intestine, acute and critical limb ischaemia) and dementia.

**Results:**

At 12 months following hospitalisation with AHRF requiring NIV, patients who developed NOAF during their admission had a statistically higher rate of death (hazard ratio (HR) 1.26, 95% CI 1.21–1.32), re-admission (HR 1.07, 95% CI 1.04–1.10), stroke (HR 1.46, 95% CI 1.27–1.68), myocardial infarction (HR 1.41, 95% CI 1.31–1.51) and the composite embolic end-point (HR 1.35, 95% CI 1.22–1.51). There was no statistically significant difference in rates of dementia (HR 1.08, 95% CI 0.97–1.21).

**Conclusion:**

The development of NOAF in AHRF requiring NIV is associated with a higher risk of mortality, readmission, stroke, myocardial infarction and composite embolism at 1 year. NOAF functions an independent indicator of poor outcomes in such patients.

## Shareable abstract

**New-onset atrial fibrillation in acute hypercapnic respiratory failure requiring noninvasive ventilation is associated with a higher mortality, readmission, stroke, myocardial infarction and composite embolism at 1 year** https://bit.ly/4eVvlz4

## Introduction

Atrial fibrillation (AF) is the most common cardiac arrhythmia worldwide and confers significant morbidity and mortality in the form of embolic stroke, heart failure and sudden death [1]. COPD is the most common chronic lung disease with an estimated worldwide prevalence of roughly 9–12% [2]. New-onset AF (NOAF) is twice as common in COPD patients as compared to patients without COPD [3] and it is estimated that roughly 20% of all COPD patients suffer with concomitant AF [4, 5]. Patients with both conditions are at an increased risk of hospitalisation and all-cause death [4, 6].

The development of AF is a multifactorial process driven by underlying risk factors. These factors contribute to atrial fibrosis and scarring, leading to adverse adaptations within the myocytes and subsequent electrical remodelling [7]. COPD leads to increased right heart pressures and a compensatory dilation of the right atrium and hypertrophy of the right ventricle. Atrial dilation is a well-recognised risk factor for AF [8]. The association between AF and COPD is not yet completely understood but COPD and AF both share common risk factors in the form of advancing age and smoking. However, COPD can also directly contribute to the onset of AF *via* hypoxia (acute or chronic) and hypercapnia. Hypoxia is associated with compensatory fluctuations in autonomic tone, pulmonary vascular pressure and cardiac haemodynamics [9]. Also, hypercapnia is associated with significantly altered atrial physiology in the form of increasing both atrial conduction time and the atrial effective refractory period in electrophysiology studies [10]. Reduced forced expiratory volume in 1 s in COPD is strongly linked to the likelihood of the development of NOAF [11].

Patients with COPD are prone to acute exacerbations requiring hospitalisation, which may be associated with hypercapnia in up to 30% of cases [12, 13]. Acute hypercapnic respiratory failure (AHRF) is associated with a 1-year mortality rate of 28% [14]. In AHRF, contemporary guidelines advocate for the use of noninvasive ventilation (NIV) [15], which is proven to reduce the need for intubation, mortality and length of hospital stay [16].

Prior data has demonstrated that AHRF is associated with the development of NOAF with an estimated prevalence of approximately 20% [17]. Whilst it has previously been recognised that AF in patients with AHRF have worse in-hospital outcomes, it is uncertain if this trend persists after discharge in the medium- and long-term [13]. To address this issue, the aim of this study utilising a global federated database of real-world data was to evaluate the long-term prognostic impact of NOAF in COPD patients hospitalised with acute respiratory failure requiring NIV.

## Methods

We utilised data from the TriNetX database, a global health research network that provides access to real-world patient data from healthcare organisations (HCOs) covering approximately 163 million individuals’ electronic medical records. Within the TriNetX database, a total of 144 health HCOs are represented from 14 different countries. The platform incorporates anonymised electronic health record data encompassing diagnoses, treatments, medications, procedures and outcomes. It allows users to construct targeted patient cohorts based on conditions, treatments and demographic information. This capability supports group comparisons, providing a robust tool for generating and validating hypotheses. Research utilising the TriNetX network has contributed to roughly ∼1400 publications between 2018–2025 (https://pubmed.ncbi.nlm.nih.gov/?term=trinetx).

All data collection, processing and transmission were conducted in compliance with all data protection laws applicable to the contributing HCOs, including the EU Data Protection Law Regulation 2016/679, the General Data Protection Regulation on the protection of natural persons with regards to the processing of personal data and the Health Insurance Portability and Accountability Act, and the US federal law which protects the privacy and security of healthcare data. The TriNetX Collaborative Networks are distributed networks and analytics are performed at the HCO with only aggregate results being surfaced and returned to the platform. Data usage and publication agreements are in place with all HCOs.

### Ethics statement

TriNetX is a global federated network that incorporates data from all participating HCOs. Each HCO ensures it has the appropriate rights, consents and approvals to share data under a business associate agreement. All data, including that from the contributing HCOs, are anonymised and used solely for research purposes, with safeguards in place to prevent the identification of individual patients. Therefore, ethical approval is not required for research conducted using these data.

### Study design

The search was conducted on 4 April 2025, with predefined inclusion and exclusion criteria based on the relevant International Classification of Diseases, Tenth Revision, Clinical Modification codes (ICD-10) to create our cohort. We utilised codes designed to identify COPD patients admitted with AHRF requiring NIV. All participants must be 1) diagnosed with COPD (ICD-10 J44) and 2) have acute respiratory failure requiring NIV within 7 days of AHRF (A09357, 5A09457, 5A09557, 5A09358, 5A09458, 5A09558, 5A0935Z, 5A0955z, 5A09459, 5A0955B, 5A0945B, 5A09359, 5A0935B and 5A09559). Patients were excluded if they had a diagnosis of AF (ICD-10 I48) prior to inclusion into the study. We searched the TriNetX database with no date restrictions to widen our search results. Eligible patients were identified and then two cohorts were created based on NOAF recorded in the patients’ medical record up to 7 days after meeting our inclusion criteria. We selected 7 days as our cut-off as it is recognised that most NOAF occurs within the first few days [18]. We aimed to capture the first episode of AF in patients with respiratory failure requiring NIV.

### Statistical analysis

The study's data and outcomes were analysed based on the index event, the time window and a list of selected outcomes. The index event is the point at which a patient enters the analysis, determined by the date the patient met the study's inclusion criteria. The index date refers to the day a patient was first recorded as meeting these criteria. The time window begins on the index event date and continues for 12 months. Normally distributed data were presented as mean±sd.

Propensity score matching (PSM) was applied to minimise differences between the cohorts and address baseline variability. We selected variables that could influence outcomes to reduce the likelihood of confounding in our results. Patients with NOAF were 1:1 propensity-score matched to patients without NOAF using logistic regression for age at index event, ethnicity (black, white and Asian), female sex, male sex, hypertension (I10), heart failure (I50), ischaemic heart disease (I20–I25), neoplasms (C00–D49), chronic kidney disease (N18), asthma (J45), peripheral vascular disease (I73), cerebral infarction (I63), pulmonary embolism (I26), deep vein thrombosis (I82.40), pulmonary fibrosis (J84.10), diabetes mellitus (E08–E13) and body mass index (Z69). These variables were chosen on the basis that they are established risk factors for cardiovascular disease/and or mortality or they were significantly different between both cohorts. The platform uses “greedy nearest-neighbour matching” with a calliper of 0.1 pooled sds and difference between propensity scores of ≤0.1. Covariate balance between groups was assessed using strictly standardised mean differences. Any baseline characteristic with a standardised mean difference between cohorts of <0.1 is considered well matched. We calculated the hazard ratio (HR) for mortality using a log-rank analysis of the propensity-score matched cohort with a 95% confidence interval (95% CI).

Following PSM, logistic regression produced hazard ratios (HR) with 95% confidence intervals for 1-year outcomes (ICD-10 codes), all-cause mortality (D: deceased), hospitalisation (1013659: hospital inpatient services), stroke (I63: cerebral infarction), myocardial infarction (I21: acute myocardial infarction), our composite embolic phenomena outcome (acquired absence of limb (Z89), acute vascular disorders of intestine (K55), acute and critical limb ischaemia (I70.2, I74)) and dementia (F03). Statistical analyses were completed using algorithms available in the TriNetX online platform which is based on R for statistical computing (v4.3.1, R Foundation for Statistical Computing, Vienna, Austria). Statistical significance was set at p<0.05.

## Results

A total of 14 262 patients out of 99 359 developed NOAF within 7 days, corresponding to an incidence of 14.9%. At baseline, the NOAF cohort was older and had a higher proportion of males. The control group had higher rates of hypertension, ischaemic heart disease and heart failure at baseline. After PSM, our cohorts were well balanced.

**FIGURE 1:**
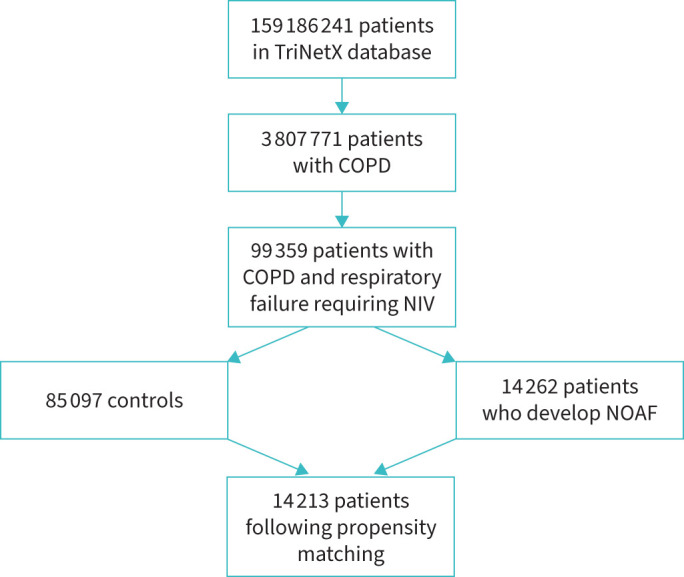
Flowchart illustrating the selection process for the study cohort. From an initial population of 159 186 241 patients in the TriNetX database, 3 807 771 patients with COPD were identified. Among these, 99 359 patients had COPD with respiratory failure requiring noninvasive ventilation (NIV). Two groups were created, as follows: 14 262 patients who developed new-onset atrial fibrillation (NOAF) and 85 097 controls. Following propensity score matching, 14 213 patients were included in the final analysis.

Table 1 shows baseline differences between both cohorts before and after PSM. Supplementary figure 1 represents our PSM matching graph. After PSM there was a total of 14 213 patients in each cohort. Median follow-up in the NOAF group was 105 (interquartile range (IQR) 50–401 days) and in the control group, 164 (IQR 60–410) days. Figure 1 demonstrates our patient selection flowchart.

**TABLE 1:** New-onset atrial fibrillation and control patients baseline characteristics before and after propensity score matching (PSM)

Characteristic	Before PSM			After PSM		
	New-onset atrial fibrillation	Control	p-value, SSMD	New-onset atrial fibrillation	Control	p-value, SSMD
**Mean±sd age at index, years**	72.3±10	68.8±11.7	<0.0001, 0.32	72±10	69.4±13	0.06, 0.02
**White, n (%)**	10 612 (74.6)	59 912 (70.7)	<0.0001, 0.09	10 611 (74.7)	10 822 (76.1)	0.0037, 0.03
**Male, n (%)**	7654 (53.8)	41 162 (48.6)	<0.0001, 0.1	7653 (53.8)	7641 (53.8)	0.9, 0.001
**Female, n (%)**	6463 (45.5)	43 068 (50.8)	<0.0001, 0.1	6463 (45.5)	6495 (45.7)	0.7, 0.0045
**Black or African American, n (%)**	1793 (12.6)	14 467 (17.1)	<0.0001, 0.1	1793 (12.6)	1694 (11.9)	0.07, 0.02
**Asian, n (%)**	277 (1.9)	1694 (2)	0.69, 0.0036	227 (1.9)	255 (1.8)	0.34, 0.01
**Essential hypertension, n (%)**	7047 (49.6)	55 591 (65.6)	<0.0001, 0.33	7047 (49.6)	7115 (50.1)	0.42, 0.0096
**Heart failure, n (%)**	5678 (39.9)	38 597 (45.5)	<0.0001	5678 (39.9)	5592 (39.3)	0.3, 0.01
**Ischaemic heart disease, n (%)**	5301 (37.3)	37 632 (44.4)	<0.0001, 0.14	5301 (37.3)	5214 (36.7)	0.29, 0.01
**Diabetes mellitus, n (%)**	4485 (31.6)	32 323 (38.1)	<0.0001, 0.14	4484 (31.5)	4464 (31.4)	0.80, 0.003
**Neoplasms, n (%)**	3733 (26.3)	29 399 (34.7)	<0.0001, 0.18	3733 (26.3)	3681 (25.9)	0.48, 0.008
**Body mass index, n (%)**	3669 (25.8)	29 482 (34.8)	<0.0001, 0.20	3669 (25.8)	3608 (25.4)	0.41, 0.0098
**Chronic kidney disease, n (%)**	3312 (23.3)	23 887 (28.2)	<0.0001, 0.11	3312 (23.3)	3177 (22.4)	0.06, 0.0226
**Asthma, n (%)**	1932 (13.6)	18 555 (21.9)	<0.0001, 0.22	1932 (13.6)	1847 (13)	0.14, 0.02
**Peripheral vascular disease, n (%)**	1512 (10.6)	12 260 (14.5)	<0.0001, 0.11	1512 (10.6)	1403 (9.9)	0.03, 0.03
**Pulmonary embolism, n (%)**	694 (4.9)	5451 (6.4)	<0.0001, 0.07	694 (4.9)	562 (4)	0.0001, 0.05
**Deep vein thrombosis, n (%)**	415 (2.9)	3495 (4.1)	<0.0001, 0.07	415 (2.9)	331 (2.3)	0.001, 0.03
**Cerebral infarction, n (%)**	643 (4.5)	5602 (6.6)	<0.0001, 0.09	643 (4.5)	541 (3.8)	0.002, 0.04
**Pulmonary fibrosis, n (%)**	563 (4)	4831 (5.7)	<0.0001, 0.08	563 (4)	503 (3.5)	0.06, 0.022

SSMD: strictly standardised mean difference. p-value is significant if ≤0.5.

**FIGURE 2:**
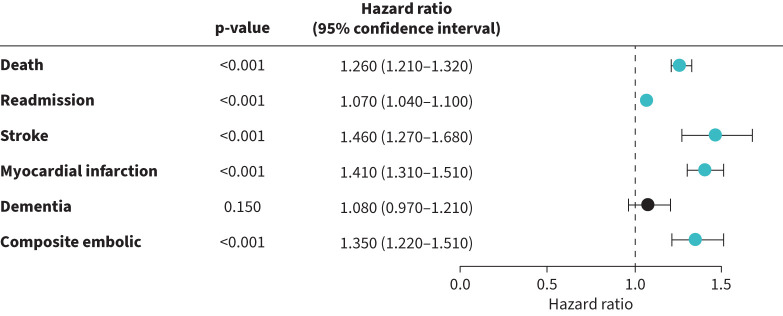
Forest plot of hazard ratios with 95% confidence intervals for clinical outcomes in the new-onset cohort compared to the control group. The black dashed vertical line at hazard ratio=1 represents the null value (no effect). p-values <0.05 indicate statistical significance.

**FIGURE 3:**
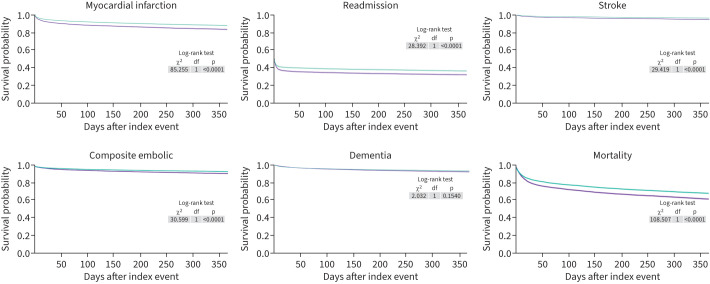
Kaplan–Meier survival curves for each outcome, stratified by the presence of new-onset atrial fibrillation. Patients with new-onset atrial fibrillation are shown in purple and those without the condition are shown in cyan.

One year after admission to hospital with AHRF requiring NIV (index event), patients who developed NOAF experienced higher mortality compared to controls (HR 1.26, 95% CI 1.21–1.32, p<0.0001), as well as higher re-admission (HR 1.07, 95% CI 1.04–1.10, p<0.0001), stroke (HR 1.46, 95% CI 1.27–1.68, p<0.0001), myocardial infarction (HR 1.41, 95% CI 1.31–1.51, p<0.0001) and the composite embolic end-point (HR 1.35, 95% CI 1.22–1.51, p<0.0001). There was no statistically significant difference in rates of dementia (HR 1.08, 95% CI 0.97–1.21, p=0.15). Table 2 demonstrates individual outcome measures and their respective E-values. Figure 2 is a forest plot representing our results. Kaplan–Meier curves for the study outcomes are shown in figure 3.

**TABLE 2:** Individual outcome measures with respective hazard ratios and E-values

Outcome	Patients with outcome in new-onset cohort group *versus* control	Hazard ratio	95% confidence interval	p-value	E-value
**Death**	4448 *versus* 3704	1.26	1.21–1.32	<0.0001	1.82
**Re-admission**	9103 *versus* 8603	1.07	1.04–1.10	<0.0001	1.22
**Stroke**	482 *versus* 347	1.46	1.27–1.68	<0.0001	2.02
**Myocardial infarction**	1684 *versus* 1270	1.41	1.31–1.51	<0.0001	2.05
**Dementia**	658 *versus* 640	1.08	0.97–1.21	0.15	Not applicable
**Composite embolic outcome**	749 *versus* 590	1.35	1.22–1.51	<0.0001	2.04

To enhance the robustness of our findings, we calculated E-values as a quantitative bias analysis to reduce the risk of unidentified residual confounding, aiding readers in interpreting our results [19]. Larger (higher numbers) E-values suggest stronger evidence against confounding as the sole explanation for our observed associations. This suggests that the results for readmission (E-values of 1.22) are more susceptible to bias as a relatively weak unmeasured confounder could explain the results. In contrast, for ischaemic stroke (2.02), myocardial infarction (2.05) and death (1.82), there would need to be a strong unmeasured confounder to explain these results.

## Discussion

In this large cohort study of ∼28 000 well-matched patients with COPD admitted with AHRF requiring NIV and without a prior history of AF, we demonstrate that NOAF is associated with significantly worse long-term clinical outcomes. At 1 year post-admission, patients who developed NOAF had higher mortality, increased risk of hospital readmission and a greater incidence of stroke, myocardial infarction and thromboembolic events.

To our knowledge, this represents the largest study to date focusing on the prognostic significance of NOAF in the context of COPD patients managed with NIV for AHRF. Our study suggests an incidence rate of NOAF in this patient population of roughly 14.9%, which is comparable to prior studies looking at the incidence of NOAF in exacerbations of COPD [17, 20]. Our results support planning of future prospective studies in critically unwell patients with AF.

NIV is a cornerstone in the management of AHRF in COPD, offering an evidence-based intervention that reduces the need for intubation and improves survival. However, NIV increases intrathoracic pressures, reducing venous return and raising right atrial and pulmonary artery pressures, potentially influencing left atrial mechanics and predisposing to arrhythmogenesis. This haemodynamic interplay, particularly in the setting of already strained cardiopulmonary physiology, may partially explain the emergence of NOAF during the acute phase of care [21].

It has long been recognised that AHRF confers poor outcomes in COPD hospitalisations. However, it was only in 2012 with the proposal of the DECAF score (Dyspnoea, Eosinopenia, Consolidation, Acidaemia and atrial Fibrillation) that the association between AF and higher mortality in COPD exacerbation was noted [13]. This score predicts in-hospital mortality in COPD exacerbations with an area under the receiver operating characteristic curve of 0.82–0.86 overall [13]. Notably, this score does not differentiate between NOAF and pre-existing AF on admission. The authors of DECAF subsequently created the NIVO (Noninvasive Ventilation Outcomes) score in 2021 to help better predict both in-hospital and 90-day mortality in patients hospitalised with exacerbations of COPD [22]. However, there are no treatment recommendations based on this score, it is simply to help guide decision-making in escalation invasive ventilation or transfer to a more closely monitored unit. Within the NIVO score, AF conferred an odds ratio for in-hospital mortality of 3.66, which is higher than any other component of the score except for eosinophilia, a time to acidaemia of >12 h and pre-admission baseline breathless during self-care (washing/dressing). This underscores the prognostic significance of AF in AHRF. However, the authors did not investigate if NOAF conferred a differential risk compared to pre-existing AF. There is also the growing recognition that AF is a heterogenous condition with distinct clinical patterns based on medical background and ethnicity that confer differential thrombotic and bleeding outcomes [23–26]. Hence it is likely that NOAF and pre-existing AF confer different prognostic risks.

Despite the prognostic importance of AF in COPD exacerbations, highlighted in scores such as DECAF and NIVO, these tools do not distinguish between pre-existing and incident AF, nor do they currently influence treatment decisions. Our findings suggest that NOAF is not merely a surrogate marker of disease severity, but an independent and actionable risk factor. Given the heterogeneity of AF and its interaction with COPD-specific pathophysiology and treatment (*e.g.*, bronchodilators, beta-blockers and antiarrhythmic agents), a nuanced approach to management is essential.

The high incidence of NOAF (∼14.9%) in this population, along with its significant prognostic implications, underscores the need for future prospective studies to better define pathophysiological mechanisms, risk stratification and tailored treatment strategies in this cohort. Importantly, our study supports the adoption of a holistic, integrated model of care for patients with coexisting COPD and AF, which includes not only anticoagulation and rhythm/rate control, but also optimisation of respiratory status, early identification of arrhythmias and careful medication reconciliation to mitigate potential iatrogenic harm.

It is well recognised that COPD affects cardiac structure and function with adaptive changes seen in the pulmonary blood vessels and both the right and left ventricles and atria [27]. Chronic hypoxia, which can be present in severe COPD, induces pulmonary vascular remodelling, pulmonary hypertension and right ventricular hypertrophy. Indeed, the severity of COPD is directly linked to the likelihood of pulmonary hypertension [28]. Prior data from ambulatory cardiac monitoring has demonstrated that COPD patients are significantly more likely to experience both atrial and ventricular arrythmias, and the prevalence of these arrythmias increases with the severity of COPD [29]. Indeed, AF in COPD is recognised to be an independent risk factor, rather than just a proxy for disease progression [30, 31].

AF management within the context of COPD is complicated by disease specific factors that limit both treatment of COPD and AF. Drugs used in the management of COPD such as methylxanthines or β2-agonists are well recognised to induce tachycardia and/or propagate arrythmia [32]. Hence these should be utilised with caution in patients with AF. Conversely, arrhythmic drugs used to control COPD such as sotalol and propafenone have the potential to provoke bronchospasm and decrease lung function. Furthermore, amiodarone is implicated in causing lung fibrosis [32]. Indeed, COPD patients with coexistent AF are more likely to be rate controlled with calcium channel blockers and digoxin compared to non-COPD patients [33]. This is despite beta blockers generally being considered generally safe with some data demonstrating a reduction in all-cause mortality in COPD patients [34]. Furthermore, COPD patients are less likely to be referred for cardioversion/ablation despite the absence of convincing evidence that these strategies are less likely to work in this patient cohort [33, 35, 36].

Our study underlines the complexity arising by the contemporary diagnosis of AF in patients with COPD. Our findings are consistent with the need for holistic and integrated care management, as promoted in the evidence-based Atrial fibrillation Better Care (ABC) pathway [37], which emphasises appropriate oral anticoagulation and patient-centred rhythm or rate-control strategies as well as the optimal management of comorbidities, including respiratory conditions. This integrated approach has been shown to be beneficial across different AF patient phenotypes and clinical settings [38–42]. Of note, a similar holistic approach concept has also been promoted by European and American AF management guidelines with the (untested) AF-CARE [43] and SOS [44] acronyms, respectively.

### Limitations

Our study has several limitations. First, this a retrospective study utilising data extracted from an electronic database (without access to individual charts) in the form of ICD-10 codes. There is significant potential for data to be incomplete, or inappropriately coded, especially as these data are collected for routine clinical practice and invoicing. Second, whilst the TriNetX database allows us to derive data for rehospitalisation, we are unable to break this down further to determine the reason for readmission to hospital. Third, statistical analysis is performed within the TriNetX in-built platform and does not allow the user access to the raw data. Furthermore, we are unable to derive the onset time of events, only that events have occurred within the observed time window. In addition, whilst we report the mean duration of follow-up, the TriNetX system lacks granular details such as exact number of patients at the end-point of the study. Importantly, the size of the TriNetX network can function as a limitation of our findings. Whilst an increasing sample size generally increases statistical power, it also makes it more likely to detect statistically significant differences when the effect size is small which may not be necessarily clinically or practically meaningful. Finally, given the observational nature of the study, we are unable to determine cause–effect relationships; and the possibility of unspecified confounders remains. For example, there are reported ethnic differences in AF epidemiology as well as AF-related outcomes such as stroke and bleeding [45–47]. Kaplan–Meier curves performed in the TriNetX platform have significant limitations that affect interpretation. The platform functions as a “black box”, offering limited insight into how time-to-event variables are defined. Researchers have no control over data cleaning, censoring mechanisms or modelling assumptions. Patient follow-up is highly variable and there is no reliable method to distinguish between true censoring and low follow-up. Patients may pass away, leave the network or simply stop receiving care within the network and these scenarios would be indistinguishable within the data. Furthermore, the visual output lacks key analytical features such as confidence intervals, censoring marks or detailed statistical outputs. Therefore, we regard our study as hypothesis generating, highlighting the need for future studies.

### Conclusions

The development of NOAF in AHRF requiring NIV is associated with a higher risk of mortality, readmission, stroke, myocardial infarction and composite embolism at 1 year. NOAF functions as an independent indicator of poor outcomes in such patients.

## Data Availability

Our data are available for review upon access to the TriNetX network.

## Supplementary material

**Please note:** supplementary material is not edited by the Editorial Office, and is uploaded as it has been supplied by the author. Supplementary material 00605-2025.SUPPLEMENT
