# Supplementary figures and images for: New-onset atrial fibrillation in patients with acute hypercapnic respiratory failure requiring noninvasive ventilation
Source: ERJ Open Res. 2025 Dec 22;11(6):00605-2025. doi: 10.1183/23120541.00605-2025 (PMC12720159; doi:10.1183/23120541.00605-2025)

# Supplementary figure 1

Propensity Score Density Function  
Before Matching

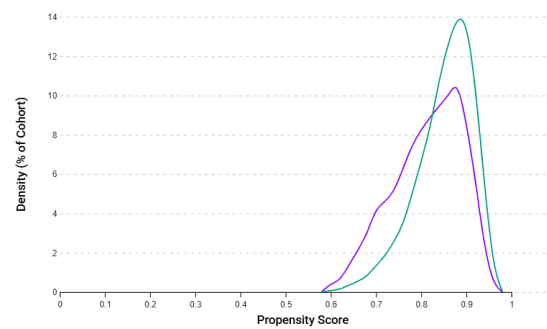

After Matching

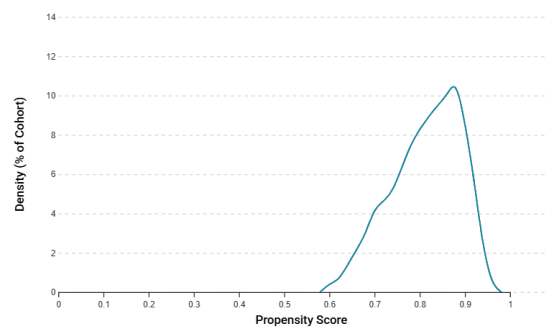

Supplement: Supplementary file 1 [file 00605-2025.SUPPLEMENT.pdf]
